# Relationship between three dietary indices and health-related quality of life among rural elderly in China: a cross-sectional study

**DOI:** 10.3389/fnut.2023.1259227

**Published:** 2023-10-19

**Authors:** Chen Yang, Peijun Liu, Wenjing Huang, Ying Zhou, Cuiping Liu, Tianlin Gao, Feng Zhong

**Affiliations:** ^1^Qingdao University School of Public Health, Qingdao, China; ^2^The Affiliated Hospital of Qingdao University, Qingdao, China; ^3^Shandong First Medical University & Shandong Academy of Medical Sciences, Jinan, China

**Keywords:** elderly, rural, dietary, quality of life, HRQOL

## Abstract

**Purpose:**

This study aimed to explore the association between health-related quality of life (HRQOL) and diet quality using three evidence-based dietary indices among older people in rural China.

**Methods:**

This cross-sectional study included 1,258 rural older people (mean age 72.32 years; 55.6% female). HRQOL was assessed using the European Five Dimension Health Scale (EQ-5D), and dietary intake was assessed using a Food Frequency Questionnaire. Three dietary scoring indices, including the Alternate Healthy Eating Index, Dietary Approaches to Stop Hypertension, and Dietary Diversity Score (DDS), were calculated to assess and analyze the relationship between these dietary indices and quality of life.

**Results:**

The EQ-5D score was 0.95 ± 0.10, and the EQ-Visual Analog Scale (VAS) score was 76.76 ± 14.44. All three groups with higher dietary indices had higher quality of life scores. After controlling for covariates in multivariate adjusted binary logistic regression analyzes, participants in the top tertile of DDS had higher quality of life scores than those in the bottom tertile. DDS was consistently associated with EQ-5D (Model 2: OR = 1.567, *p* = 0.001; Model3: OR = 1.351, *p* = 0.044) and EQ-VAS (Model 2: OR = 1.830, *p* < 0.001; Model 3: OR = 1.383, *p* = 0.047), significantly different from the other groups.

**Conclusion:**

Older people in rural China who adhere to various foods experience a better quality of healthy life.

## 1. Introduction

As the world’s population grows older, aging is becoming a serious social problem. By 2050, the number of people over 60 years worldwide will double from 1.4 billion in 2015 to 2.1 billion (1). According to the latest census results in China, 18.70 and 13.50% of the total population are aged ≥60 years old and ≥ 65 years old, respectively. The township population accounts for 36.11% of the total population (2). Due to urbanization, most young people start to work and live in the cities, while the older people remain in the rural areas (3). As a result, the quality of life of the elderly living in rural areas has attracted attention (4, 5). Older people in rural China generally have inadequate nutritional intake (6), which is extremely detrimental to their nutritional health (7). The risk of malnutrition among Chinese elders is 12.6% (8). Several cross-sectional and cohort studies have shown negative associations between healthy and adequate nutritional status and chronic disease risk (9–11). Additionally, nutrition is closely related to the health-related quality of life (HRQOL) of rural older people.

HRQOL is a multidimensional indicator of overall health. It can be defined as a composite confounding criterion of social relationships, personal emotions, independence, etc., significantly related to the quality of life consequences of an individual’s health status. Many scales can be used to evaluate the quality of a healthy life, such as the Brief Health Questionnaire, the World Health Organization (WHO) Quality of Life Scale (WHOQOL), and the Quality of Well-Being Scale (QWB) (12). Quality of life assessment tools for older adults include the 36-item Brief Health Status Inventory (SF-36), the European Five Dimension Health Scale (EQ-5D), and the WHOQOL. The EQ-5D has feasibility properties in the elderly population (13). The Chinese version of the EQ-5D scale has been widely used in China and has good reliability and validity (14). Therefore, we chose the EQ-5D to assess the quality of life of older people in China.

Adequate nutrition is an essential component of healthy aging (15). It is well known that the nutritional status of older people is critical to health. However, with increasing age and declining physical functions, older people may develop a loss of appetite and decreased ability to chew (16). This can lead to weight loss and malnutrition. Moreover, the income level of older people affects their nutritional status: research has shown that financial hardship is associated with nutritional problems (17). Most older people in rural China have low economic incomes, limiting their ability to afford a high-quality diet or maintain good nutrition. It is, thus, of significant interest to evaluate the dietary quality of older Chinese people in rural areas.

Diet Quality Index (DQI) is a common index to evaluate diet quality, such as the Mediterranean (MED) (18), the Alternate Healthy Eating Index (AHEI) (19), the Dietary Approaches to Stop Hypertension (DASH) (20), and the Dietary Diversity Score (DDS) (21). Existing studies have mostly analyzed the correlation between one DQI or different DQIs and different diseases (22–25). A large study investigated the association of diet quality, assessed by the AHEI, MEDAS, and DDS score, with health status (all-cause mortality, cardiovascular disease mortality or morbidity, cancer mortality or morbidity, type 2 diabetes, and neurodegenerative disease risk) (26). However, there are few studies on the relationship between quality of life and DQI in older people, especially in rural Chinese older people (27). In this study, three dietary index scores were selected to assess the quality of life of older people in rural China.

## 2. Materials and methods

### 2.1. Study design and study population

This cross-sectional study included 1,258 people (≧65 years) living in rural China. All participants in the study were volunteers who signed informed consent forms.

**Figure 1:**
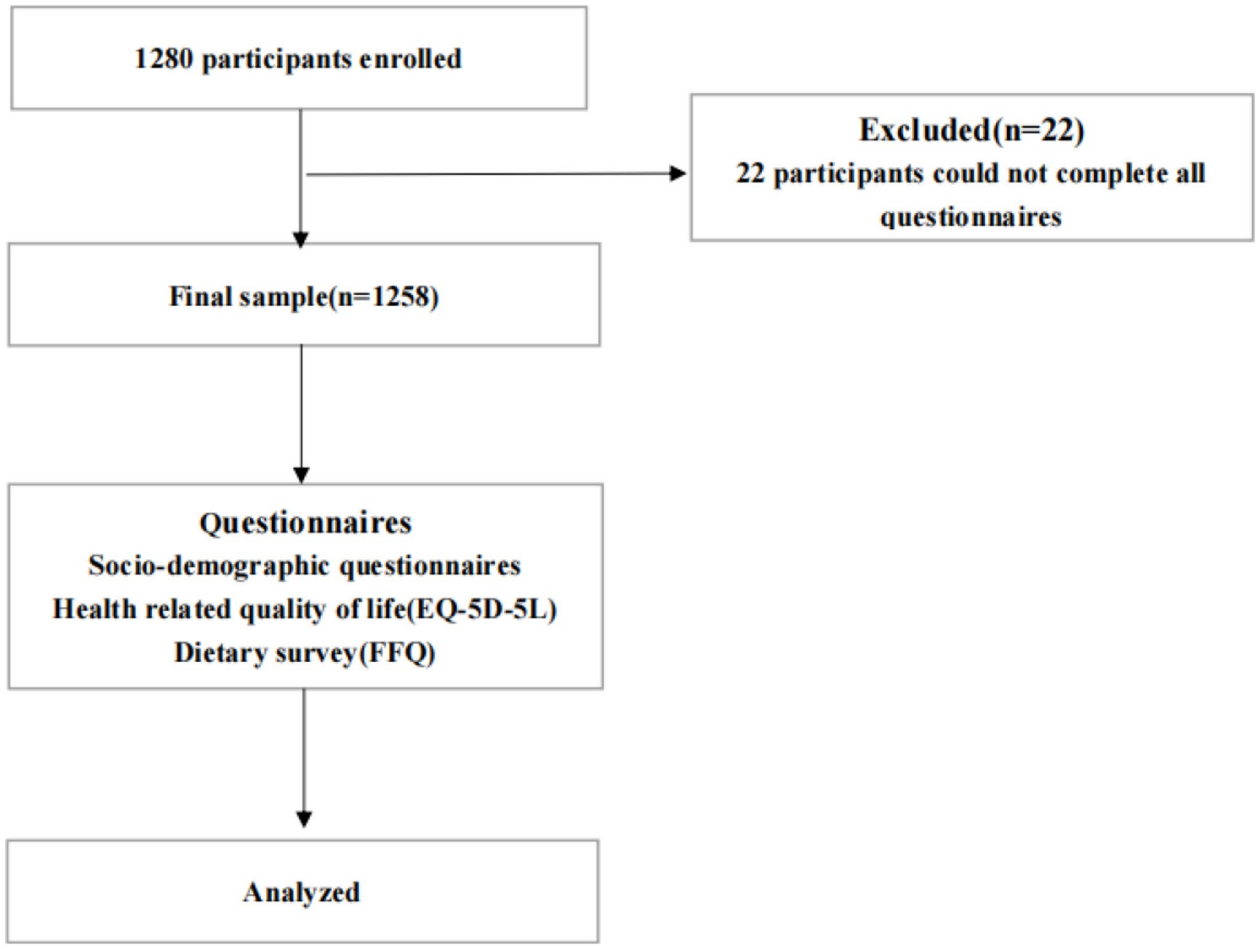
Flow chart for the course of study.

As shown in Figure 1, 1,280 older people were invited to this study (94.4% participation rate), and 22 participants were excluded because they could not complete all questionnaires. Therefore, data from 1,258 older people (mean age 72.32 ± 6.00 years; 55.6% female) were analyzed.

### 2.2. Dietary survey

The Food Frequency Questionnaire (FFQ, containing 97 food items), which has been used in studies of Chinese residents with good results and high reliability, was used to measure the dietary intake of respondents in the past 3 months (28, 29). Based on the latest version of the Chinese food composition table, we constructed different nutritional variables such as whole grains, vegetables, fruits, red meat, sugary drinks, alcohol, trans fatty acids, omega fatty acids (EPA + DHA), and sodium.

### 2.3. Dietary quality indices

Using the dietary intake data derived from the FFQ, we selected the following DQIs: AHEI, DASH, and DDS. The three dietary index scores were derived based on the protocols of Stephanie (9), Teresa (30), and Jin (31), respectively.

The AHEI score consisted of 11 items. We assigned a maximum score of 10 to each item if the recommended intake was achieved and a minimum score of 0 for minimum intake. The total score ranged from 0 to 110, depending on the level of intake.

The DASH score was based on the intake level of all people, regardless of the total intake in the entire population. Each food category was divided into quintiles and there were eight food groups in total. For foods beneficial to health, such as fruits, vegetables, nuts, and legumes, we assigned a categorical score: quintile 1 and quintile 5 were scored 1 and 5, respectively. However, for sugary drinks and red meat, the scale was reversed, and quintile 1 was assigned a score of 5. The total DASH score ranged from 8 to 40 points.

The DDS score was developed by Kant et al. (32). We combined the Chinese Dietary Guidelines and the dietary recommendations of the Chinese Nutrition Association to change the DDS score to a more suitable scale for China (31). Foods were divided into nine categories. The food groups consumed by the study subjects in a week were investigated. One point was assigned to each food group consumed, regardless of the number of times and the amount of intake, and the same food group was not repeatedly scored. The total possible score was nine.

### 2.4. HRQOL

The EuroQol Five-Dimensional Scale (EQ-5D) consists of two parts: the Five-Dimensional Health Description System and the Visual Analog Scale (EQ-VAS) (33, 34). The five-dimensional health description system includes five dimensions: mobility, self-care, usual activities, pain/discomfort, and anxiety/depression. Each dimension corresponds to five levels, describing 3,125 health states (35). The EQ-VAS is a visual scale from 0 (worst health self-rated status) to 100 (best health self-rated status) and is an individual’s assessment of their health status on the day of measurement. This study used the utility value score system developed by Luo et al. (36) to evaluate the health utility index of the Chinese population. The dimensions and degrees were multiplied together, and the results were summed to give a health utility value for each individual. The EQ-5D health utility score ranges from −0.391 to 1: the higher the score, the better the health status.

### 2.5. Statistical analysis

Based on the distribution of each dietary index score and number of participants, we divided participants into three groups: T1, T2, and T3. However, due to the concentration of DASH and DDS scores, we grouped the concentrated values as one category to complete the grouping—many participants in the DASH and DDS groups had the same score value and could not be separated into the two groups, resulting in differences in the number of participants in each group. Chi-square tests were used to compare categorical variables between quartiles of dietary pattern scores. For continuous variables, we first tested the normality assumption using the Shapiro–Wilk test. Non-normally distributed variables were normalized using a logarithmic transformation. One-way analysis of variance was then used to compare continuous variables between dietary pattern scores. HRQOL was categorized into a high and a low group according to its distribution and analyzed using binary logistic regression. The multivariate adjustment model employed binary logistic regression: Model 1 did not adjust for any factors; Model 2 adjusted for gender, age, and body mass index; Model 3 additionally adjusted for smoking, alcohol consumption, chronic illness, cognitive status, residence, income, and education (each factor that had an impact on the quality of life of older people was selected based on a one-way analysis of the Supplementary Table).

All data analyzes were performed using the Statistical Package for Social Sciences software (version 25), and all significance levels were two-sided *p*-values <0.050. Categorical data were expressed as percentages. Continuous variables were expressed as arithmetic means and log-transformed variables at 95%.

## 3. Results

### 3.1. Three different HRQOLs with basic participant characteristics

A total of 1,258 participants were included in this study; 55.6% (*n* = 699) of the participants were female, with a mean age of 72.32 ± 6.00 years. T3 in the DASH group had the lowest percentage of smokers (23.7%) and the lowest percentage of hypertensive patients (33.7%) and T3 group of DDS had the lowest proportion of malnutrition (16.4%) and the lowest proportion of MCI (14.8%).Three-quarters (75.4%) of the participants were married, and 1,056 (83.9%) lived with their families. Participants in the highest group (T3) of each diet index score were more likely to be educated, married, live with their families, be highly active, have a higher income, and have good nutritional and cognitive status (Table 1).

**Table 1:** Characteristics of study participants.

	Total	AHEI			DASH			DDS		
	(*n* = 1,258)	T1 (*n* = 443)	T2 (*n* = 399)	T3 (*n* = 416)	T1 (*n* = 259)	T2 (*n* = 510)	T3 (*n* = 489)	T1 (*n* = 471)	T2 (*n* = 299)	T3 (*n* = 488)
*Age*	72.32 ± 6.00	72.99 ± 6.23	72.53 ± 6.00	71.41 ± 5.67	72.80 ± 6.14	72.51 ± 6.235	71.88 ± 5.67	73.06 ± 6.44	72.15 ± 5.80	71.72 ± 5.63
*BMI (%)*										
Underweight	39 (3.1)	17 (3.8)	14 (3.5)	8 (1.9)	9 (3.5)	19 (3.7)	11 (2.2)	20 (4.2)	9 (3.0)	10 (2.0)
Normal	406 (32.3)	143 (32.3)	130 (32.6)	133 (32.0)	96 (37.1)	168 (32.9)	142 (29.0)	163 (34.6)	95 (31.8)	148 (30.3)
Overweight	625 (49.7)	217 (49.0)	192 (48.1)	216 (51.9)	116 (44.8)	249 (48.8)	260 (53.2)	222 (47.1)	148 (49.5)	255 (52.3)
Obesity	188 (14.9)	66 (14.9)	63 (15.8)	59 (14.2)	38 (14.7)	74 (14.5)	76 (15.5)	66 (14.0)	47 (15.7)	75 (15.4)
*Education (%)*										
Illiteracy	575 (45.7)	230 (51.9)	183 (45.9)	162 (38.9)	123 (47.5)	266 (52.2)	186 (38.0)	257 (54.6)	149 (49.8)	169 (34.6)
Primary school	450 (35.8)	149 (33.6)	142 (35.6)	159 (38.2)	91 (35.1)	161 (31.6)	198 (40.5)	152 (32.3)	101 (33.8)	197 (40.4)
Secondary school	233 (18.5)	64 (14.4)	74 (18.5)	95 (22.8)	45 (17.4)	83 (16.3)	105 (21.5)	62 (13.2)	49 (16.4)	122 (25.0)
*Married (%)*										
Single	17 (1.4)	6 (1.4)	3 (0.8)	8 (1.9)	5 (1.9)	9 (1.8)	3 (0.6)	9 (1.9)	2 (0.7)	6 (1.2)
married	949 (75.4)	320 (72.2)	295 (73.9)	334 (80.3)	185 (71.4)	369 (72.4)	395 (80.8)	343 (72.8)	220 (73.6)	386 (79.1)
Widowed and divorced	292 (23.2)	117 (26.4)	101 (25.3)	74 (17.8)	69 (26.6)	132 (25.9)	91 (18.6)	119 (25.3)	386 (79.1)	96 (19.7)
*Residence (%)*										
alone	202 (16.1)	76 (17.2)	65 (16.3)	61 (14.7)	42 (16.2)	89 (17.5)	71 (14.5)	79 (16.8)	59 (19.7)	64 (13.1)
With family	1,056 (83.9)	367 (82.8)	334 (83.7)	355 (85.3)	217 (83.8)	421 (82.5)	418 (85.5)	392 (83.2)	240 (80.3)	424 (86.9)
*Tobacco smoking (%)*										
Yes	313 (24.9)	115 (26.0)	90 (22.6)	108 (26.0)	80 (30.9)	117 (22.9)	116 (23.7)	110 (23.4)	70 (23.4)	133 (27.3)
No	945 (75.1)	328 (74.0)	309 (77.4)	308 (74.0)	179 (69.1)	393 (77.1)	373 (76.3)	361 (76.6)	229 (76.6)	355 (72.7)
*Alcohol consumption (%)*										
Yes	334 (26.6)	102 (23.0)	94 (23.6)	138 (33.2)	75 (29.0)	126 (24.7)	133 (27.2)	99 (21.0)	77 (25.8)	158 (32.4)
No	924 (73.4)	341 (77.0)	305 (76.4)	278 (66.8)	184 (71.0)	384 (75.3)	356 (72.8)	372 (79.0)	222 (74.2)	330 (67.6)
*Annual income (%)*										
≤3,000	236 (18.8)	119 (26.9)	75 (18.8)	42 (10.1)	30 (11.6)	126 (24.7)	80 (16.4)	100 (21.2)	63 (21.1)	73 (15.0)
>3,000	1,022 (81.2)	324 (73.1)	324 (81.2)	374 (89.9)	229 (88.4)	384 (75.3)	409 (83.6)	371 (78.8)	236 (78.9)	415 (85.0)
*Hypertension (%)*										
Yes	444 (35.3)	156 (35.2)	133 (33.3)	155 (37.3)	87 (33.6)	192 (37.6)	165 (33.7)	164 (34.8)	94 (31.4)	186 (38.1)
No	814 (64.7)	287 (64.8)	266 (66.7)	261 (63.7)	172 (66.4)	318 (62.4)	324 (66.3)	307 (65.2)	205 (68.6)	302 (61.9)
*Diabetes (%)*										
Yes	235 (18.7)	75 (16.9)	84 (21.1)	76 (18.3)	29 (11.2)	52 (10.2)	63 (12.9)	48 (10.2)	31 (10.4)	65 (13.3)
No	1,023 (81.3)	368 (83.1)	315 (78.9)	340 (81.7)	230 (88.8)	458 (89.8)	426 (87.1)	423 (89.8)	268 (89.6)	423 (86.7)
*Activity time (%)*										
<2 h	373 (29.7)	145 (32.7)	124 (31.1)	104 (25.0)	86 (33.2)	156 (30.6)	131 (26.8)	170 (36.1)	96 (32.1)	107 (21.9)
≥2 h	885 (70.3)	298 (67.3)	275 (68.9)	312 (75.0)	173 (66.8)	354 (69.4)	358 (73.2)	301 (63.9)	203 (67.9)	381 (78.1)
*Nutrition (%)*										
Health	1,010 (80.3)	364 (82.2)	313 (78.4)	333 (80.0)	220 (84.9)	387 (75.9)	403 (82.4)	371 (78.8)	231 (77.3)	408 (83.6)
Malnutrition	248 (19.7)	79 (17.8)	86 (21.6)	83 (20.0)	39 (15.1)	123 (24.1)	86 (17.6)	100 (21.2)	68 (22.7)	80 (16.4)
*Cognition (%)*										
Health	944 (75.0)	308 (69.5)	304 (76.2)	332 (79.8)	185 (71.4)	364 (71.4)	395 (80.8)	313 (66.5)	215 (71.9)	416 (85.2)
MCI	314 (25.0)	135 (30.5)	95 (23.8)	84 (20.2)	74 (28.6)	146 (38.6)	94 (19.2)	158 (33.5)	84 (28.1)	72 (14.8)

BMI, body mass index; AHEI, Alternate Healthy Eating Index; DASH, Dietary Approaches to Stop Hypertension; DDS, Dietary Diversity Score.

### 3.2. Univariate analysis of different dietary quality indices on HRQOL

The EQ-5D and EQ-VAS scores were 0.95 ± 0.10 and 76.76 ± 14.44, respectively (Table 2). Across the three different dietary index scores, the T3 group had the highest EQ-5D and EQ-VAS scores (Table 2). The EQ-5D score in T3 of AHEI was 0.96 ± 0.08, DASH was 0.95 ± 0.09, and DDS was 0.96 ± 0.08; the EQ-VAS score for AHEI was 78.88 ± 15.11, DASH was 78.59 ± 14.55, and DDS was 80.06 ± 13.10. The number of people with health problems was lowest in the highest scoring group (T3) on the dimensions assessing quality of life. There were differences in self-care (*p* = 0.005) and anxious/depression (*p* = 0.012) scores by levels of AHEI. By contrast, only self-care scores (*p* = 0.009) varied by levels of DASH. Meanwhile, scores in all dimensions (mobility: *p* = 0.001, self-care: *p* < 0.001, pain/discomfort: *p* = 0.002, anxious/depression: *p* = 0.035) differed by levels of DDS.

**Table 2:** Univariate analysis of three dietary index scores and HRQOL.

	Mobility *n* (%)	Self-care *n* (%)	Usual Activities *n* (%)	Pain/discomfort *n* (%)	Anxious/Depression *n* (%)	EQ-5D utility value Mean ± SD	EQ-VAS score Mean ± SD
*Total*	138 (11.0)	116 (9.2)	158 (12.6)	370 (29.4)	133 (10.6)	0.95 ± 0.10	76.76 ± 14.44
*AHEI*							
T1	60 (13.5)	55 (12.4)	68 (15.3)	131 (29.6)	57 (12.9)	0.94 ± 0.10	76.24 ± 13.08
T2	43 (10.8)	36 (9.0)	49 (12.3)	126 (31.6)	47 (11.8)	0.94 ± 0.12	75.78 ± 14.98
T3	35 (8.4)	25 (6.0)	41 (9.9)	113 (27.2)	29 (7.0)	0.96 ± 0.08	78.88 ± 15.11
*χ*^2^ /Z	5.805	10.544	5.938	1.921	8.788	231.189	54.514
*P*	0.055	0.005	0.051	0.383	0.012	0.004	0.001
*DASH*							
T1	34 (13.1)	27 (10.4)	38 (14.7)	66 (25.5)	31 (12.0)	0.95 ± 0.10	76.81 ± 15.58
T2	59 (11.6)	59 (11.6)	69 (13.5)	158 (31.0)	59 (11.6)	0.94 ± 0.11	75.49 ± 13.56
T3	45 (9.2)	30 (6.1)	51 (10.4)	146 (19.9)	43 (8.8)	0.95 ± 0.09	78.59 ± 14.55
*χ*^2^ /Z	2.986	9.370	3.509	2.577	2.707	214.069	59.791
*P*	0.225	0.009	0.173	0.276	0.258	0.034	<0.001
*DDS*							
T1	66 (14.0)	64 (13.6)	75 (15.9)	155 (32.9)	66 (14.0)	0.93 ± 0.12	74.88 ± 14.86
T2	39 (13.0)	20 (6.7)	41 (13.7)	91 (30.4)	30 (10.0)	0.94 ± 0.10	75.20 ± 15.00
T3	33 (6.8)	32 (6.6)	42 (8.6)	124 (25.4)	37 (7.6)	0.96 ± 0.08	80.06 ± 13.10
*χ*^2^ /Z	14.628	17.157	12.159	6.689	10.604	247.932	74.400
*P*	0.001	<0.001	0.002	0.035	0.005	<0.001	<0.001

AHEI, Alternate Healthy Eating Index; DASH, Dietary Approaches to Stop Hypertension; DDS, Dietary Diversity Score.

### 3.3. Multifactorial effects of different dietary quality indices on HRQOL

Controlling for covariates in multivariate adjusted binary logistic regression analyzes, participants in the top tertile of DDS had higher quality of life scores than those in the bottom tertile (Tables 3, 4). The higher the DDS score, the greater the chance that the EQ-5D (Model 2: OR = 1.567, *p* = 0.001; Model 3: OR = 1.351, *p* = 0.044) will be in a high classification, which is similar to the trend for the EQ-VAS (Model 2: OR = 1.830, *p* < 0.001; Model3: OR = 1.383, *p* = 0.047). The EQ-VAS (Model 2: OR = 0.694, *p* = 0.041; Model 3: OR = 0.636, *p* = 0.016) was negatively associated with quality of life in the T2 score group of DASH. No significant association was found between higher AHEI scores and HRQOL after adjusting for covariates (*p* > 0.05).

**Table 3:** Estimated parameters of the EQ-5D multifactor analysis and mixed model.

		Model1			Model2			Model3		
		OR	95% CI	*p*	OR	95% CI	*p*	OR	95% CI	*p*
AHEI	T1	1.0			1.0			1.0		
	T2	1.092	(0.825–1.445)	0.537	1.058	(0.795–1.406)	0.700	0.989	(0.737–1.327)	0.940
	T3	1.298	(0.980–1.719)	0.069	1.177	(0.883–1.570)	0.266	1.069	(0.790–1.447)	0.664
DASH	T1	1.0			1.0			1.0		
	T2	0.944	(0.690–1.290)	0.715	0.999	(0.726–1.375)	0.995	1.053	(0.756–1.467)	0.759
	T3	1.016	(0.741–1.394)	0.919	1.010	(0.731–1.393)	0.954	0.951	(0.681–1.329)	0.770
DDS	T1	1.0			1.0			1.0		
	T2	1.123	(0.834–1.512)	0.445	1.108	(0.819–1.501)	0.505	1.001	(0.730–1.372)	0.996
	T3	1.646	(1.259–2.152)	<0.001	1.567	(1.191–2.060)	0.001	1.351	(1.008–1.811)	0.044

AHEI, Alternate Healthy Eating Index; DASH, Dietary Approaches to Stop Hypertension; DDS, Dietary Diversity Score; OR, odds ratio; 95% CI, 95% confidence interval.

Model 1: No adjustment for any factor; Model 2: Adjusts for gender, age, BMI; Model 3: Adds adjustments for smoking, alcohol consumption, chronic illness, cognitive status, residence, income, education.

**Table 4:** Estimated parameters of the EQ-VAS multifactor analysis and mixed model.

		Model1			Model2			Model3		
		OR	95% CI	*p*	OR	95% CI	*p*	OR	95% CI	*p*
AHEI	T1	1.0			1.0			1.0		
	T2	0.931	(0.678–1.279)	0.660	0.910	(0.661–1.254)	0.566	0.885	(0.633–1.237)	0.474
	T3	1.408	(1.044–1.900)	0.025	1.310	(0.966–1.776)	0.083	1.272	(0.919–1.761)	0.147
DASH	T1	1.0			1.0			1.0		
	T2	0.669	(0.473–0.944)	0.022	0.694	(0.489–0.985)	0.041	0.636	(0.441–0.918)	0.016
	T3	1.171	(0.841–1.631)	0.349	1.158	(0.828–1.620)	0.392	0.990	(0.695–1.409)	0.953
DDS	T1	1.0			1.0			1.0		
	T2	1.038	(0.731–1.474)	0.836	1.033	(0.725–1.472)	0.857	0.880	(0.604–1.282)	0.507
	T3	1.898	(1.415–2.521)	<0.001	1.830	(1.364–2.454)	<0.001	1.383	(1.005–1.903)	0.047

AHEI, Alternate Healthy Eating Index; DASH, Dietary Approaches to Stop Hypertension; DDS, Dietary Diversity Score; OR, odds ratio; 95% CI, 95% confidence interval.

Model 1: No adjustment for any factor; Model 2: Adjusts for gender, age, BMI; Model 3: Adds adjustments for smoking, alcohol consumption, chronic illness, cognitive status, residence, income, education.

## 4. Discussion

This study applied the European Five-Dimensional Health Scale (EQ-5D) to investigate the quality of life in a sample of older people in rural Chinese. We found that most of the older people in rural areas were farmers, and due to the low economic level and degenerative changes in old age, they were prone to various health problems. Finding ways to improve their quality of life is a difficult but urgent issue.

The EQ-5D health utility score was 0.95, similar to the Chinese population standard of EQ-5D-5L (0.946) (37). By contrast, the EQ-5D health utility score was higher than the score (0.94) found by Yang et al. (33). Additionally, the score in the study was higher than that of students in the United Kingdom (UK) (0.90 ± 0.167) (38) and Canada (0.89 ± 0.14) (39), and the score in older people in Vietnam (0.80 ± 0.20) (40). These findings suggest that older people in rural China have a higher quality of life. In a survey of older people in five cities in China, the EQ-5D health utility score was close to 1, and the local older people had better quality of life. The EQ-VAS score was based on the self-perception and health evaluation of the study participants on the day of assessment. The EQ-VAS score in this study was 76.97, which was lower than that of students in the United Kingdom (38) and Canada (39) and higher than that of older people in Vietnam (40). One possible reason is that, due to cultural traditions, Chinese people were more reluctant to report health problems than Western populations and lacked confidence in their health status (41, 42).

As we predicted, older adults who adhered to all three dietary index scores at univariate analysis had higher HRQOL scores. In particular, the group with the highest dietary pattern score had the highest HRQOL. Older adults typically experience a decline in physical and mental abilities, including mobility and digestion, putting them at higher risk for malnutrition (43, 44). This indicates that compliance with dietary patterns leads to higher quality of life.

The AHEI did not show any correlation with HRQOL after multifactor adjustment, so we speculated that the AHEI may not be suitable for the dietary patterns and habits of older people in rural China.

The DASH dietary index score was associated with the EQ-VAS after multifactorial analysis. The T2 group showed a negative correlation with HRQOL (OR < 1): according to our analysis of the scores, the groups with greater differences were in the fruit category and red meat. Participants in the T2 group consumed more red meat, which may have been associated with lower EQ-VAS scores. More importantly, because of the concentration of scores, only one population was scored in the T2 group, which was representative. This needs to be explored in further studies. This might be related to the specific dietary structure of older people in rural China, which only suggests that the DASH and AHEI dietary patterns were not suitable for the dietary patterns of older people living in rural China and does not indicate a lack of association between such diets and quality of life.

DDS is a simple and efficient way to assess diet quality as an indicator of nutritional and health status (35). DDS was used to assess nutritional adequacy and overall diet quality and was considered a key indicator of high diet quality in different populations (25, 45). The higher the DDS dietary pattern score, the higher the HRQOL score of older adults, which is consistent with the results of previous studies (46, 47). Previous studies have suggested that higher DDS scores in Chinese adults may be associated with higher protein intake (48), which may improve the risk of malnutrition in older adults. After multifactorial analysis, we found that adherence to DDS dietary index scores after adjusting for other confounders had a better effect on HRQOL older adults in rural China. The DDS dietary index score performed well in EQ-5D and EQ-VAS scores in the high-scoring subgroup. This indicates that adherence to the DDS dietary pattern improves the quality of life and life experience of older people in rural China. This was similar to the findings of Poorrezaeian (49), which illustrated that DDS is a protective factor against depression in older adults, with each unit increase in DDS associated with a 39% reduction in the risk of major depression. Mina Poorrezaeian (50) also found that anxiety scores were significantly lower in people with high dietary diversity than in those with low dietary diversity, and the two were negatively correlated. This further suggests that DDS may be a protective factor for older people’s mental quality of life and that adherence to DDS can enhance older people’s self-rated quality of life (EQ-VAS).

Meanwhile, vegetables and fruits are the main sources of antioxidants, and the intake of diverse foods may increase antioxidant capacity (48). Moreover, diverse food intake may promote healthier gut flora (51). Studies have demonstrated a positive correlation between dietary diversity and healthy gut microbial stability, which may all contribute to the fact that a diverse diet may improve the quality of life in older adults. Dietary fiber, phenolic compounds, and carotenoids, which are abundant in vegetables and fruits, also reduce the level of inflammation in the body (52). Increased inflammation is thought to be a potential mechanism for the development of mental illness (53). This suggests that adherence to DDS not only increases HRQOL scores in older adults but may also increase satisfaction with life through the modulation of mental health in older adults.

The energy and nutrient intake among older Chinese adults is inadequate, and most older people are at high risk of nutritional deficiencies, especially those living in rural areas with lower levels of education and low household income (54). During our survey, we learned that most older people in rural areas still work on the farm and eat the food they grow daily. The diets of rural Chinese older adults have a single food intake and a carbohydrate-based diet with an insufficient intake of fruits and vegetables. Therefore, a DDS dietary index score that increases food diversity will increase the intake of different nutrients for rural older people. Additionally, consuming different types of food is associated with greater psychological comfort to older people due to problems such as swallowing and will give them a sense of good appetite and a strong body.

We hope future researchers will focus more on older people in rural areas, where their intake of a single food type and poor education predispose them to neglect. Owing to urbanization and the declining physical condition of older adults, it is crucial that they receive more attention and support from society for a healthier and thriving old age.

## 5. Limitations

This study was cross-sectional. Consequently, more specific reasons and results cannot be derived. Due to the older age and the lower educational levels, older adults in rural areas may express their physical condition poorly using questionnaires. More objective indicators should, therefore, be used for analysis. To better represent the national level of older people, samples should be taken from multiple regions and analyzed. Additionally, the diet of older people is best investigated in four different seasons to reflect their dietary status better. Although we have adjusted for multiple confounding factors, we could not adjust for all possible confounding factors.

## 6. Conclusion

This study adds to the evidence that adherence to the DDS dietary pattern is associated with a higher quality of life. The diets of older people in rural China are mainly carbohydrate-based, with a single type of food intake and a low intake of fruit, vegetables, and milk. We found a positive association between DDS and quality of life. Therefore, we recommend that older people should consume a variety of foods daily. With a balanced intake of various nutrients, increasing the intake of more food groups, especially fruits and vegetables, is likely to improve the quality of life of older people in rural areas.

Finally, society and the government should pay more attention to the health status and quality of life of older people. Not only dietary issues but also psychological and social issues can affect the quality of life of older people in their later years, which all require the attention of society.

## Data availability statement

The original contributions presented in the study are included in the article/Supplementary material, further inquiries can be directed to the corresponding author.

## Author contributions

CY: Data curation, Formal analysis, Investigation, Software, Writing – original draft, Writing – review & editing. PL: Investigation, Writing – original draft. WH: Writing – review & editing. YZ: Data curation, Investigation, Writing – original draft. CL: Writing – review & editing. TG: Data curation, Formal analysis, Writing – review & editing. FZ: Data curation, Formal analysis, Funding acquisition, Investigation, Project administration, Resources, Supervision, Writing – review & editing.
