# Supplementary material for: Relationship between three dietary indices and health-related quality of life among rural elderly in China: a cross-sectional study
Source: Front Nutr. 2023 Oct 19;10:1259227. doi: 10.3389/fnut.2023.1259227 (PMC10624226; doi:10.3389/fnut.2023.1259227)
Supplement: Supplementary file 1 [file Table_1.DOCX]

Supplementary Material

The relationship between three dietary indices and health-related quality of life among rural elder in China: A cross-sectional study

Chen Yang, Peijun Liu, Wenjing Huang, Ying Zhou, Cuiping Liu, Tianlin Gao, Feng Zhong^*^

*** Correspondence:** Feng Zhong: email [zhfeng@qdu.edu.cn](mailto:zhfeng@qdu.edu.cn)

# Supplementary Table

**Supplementary table** **Univariate analysis of quality of life and characteristics of participants**

|  | **Mobility**  **n (%)** | **Self-care**  **n (%)** | **Usual Activities**  **n (%)** | **Pain/discomfort**  **n (%)** | **Anxious/Depression**  **n (%)** | **EQ-5D utility value**  **Mean±SD** | **EQ-VAS score**  **Mean±SD** |
| --- | --- | --- | --- | --- | --- | --- | --- |
| **Gender** |  |  |  |  |  |  |  |
| Men | 52(9.3) | 60(10.7) | 67(12.0) | 127(22.7) | 53(8.5) | 0.95±0.10 | 78.57±14.45 |
| Women | 86(12.3) | 56(8.0) | 91(13.0) | 243(34.8) | 80(11.4) | 0.94±0.10 | 75.69±14.31 |
| χ 2 /Z | 2.86 | 2.75 | 0.30 | 21.70 | 1.27 | －3.83 | －4.21 |
| P | 0.09 | 0.09 | 0.58 | ＜0.001 | 0.26 | ＜0.001 | ＜0.001 |
| **Age** |  |  |  |  |  |  |  |
| 65-74 | 73(8.6) | 54(6.3) | 88(10.3) | 228(26.7) | 86(10.1) | 0.95±0.09 | 77.70±14.01 |
| ≥75 | 65(16.0) | 62(15.3) | 70(17.3) | 142(35.1) | 47(11.6) | 0.93±0.12 | 75.42±15.20 |
| χ 2 /Z | 15.78 | 26.44 | 12.14 | 9.18 | 0.67 | －3.77 | －2.81 |
| P | ＜0.001 | ＜0.001 | ＜0.001 | 0.002 | 0.41 | ＜0.001 | 0.005 |
| **Education** |  |  |  |  |  |  |  |
| Illiteracy | 77(13.4) | 62(10.8) | 86(1.5) | 202(35.1) | 69(12.0) | 0.93±0.11 | 73.99±15.26 |
| Primary school | 46(10.2) | 40(8.9) | 55(12.2) | 121(26.9) | 38(8.4) | 0.95±0.09 | 78.82±12.78 |
| Secondary school | 15(6.4) | 14(6.0) | 17(7.3) | 47(20.2) | 26(11.2) | 0.96±0.09 | 80.73±13.95 |
| χ 2 /Z | 8.61 | 4.61 | 8.93 | 20.01 | 3.48 | 20.07 | 49.53 |
| P | 0.01 | 0.10 | 0.01 | ＜0.001 | 0.176 | ＜0.001 | ＜0.001 |
| **residence** |  |  |  |  |  |  |  |
| alone | 28(13.9) | 23(11.4) | 27(13.4) | 70(34.7) | 33(16.3) | 0.93±0.11 | 75.22±15.76 |
| With family | 110(10.4) | 93(8.8) | 131(12.4) | 300(28.4) | 100(9.5) | 0.95±0.10 | 77.30±14.15 |
| χ 2 /Z | 2.06 | 1.35 | 0.14 | 3.19 | 9.46 | －2.79 | －1.75 |
| P | 0.15 | 0.25 | 0.71 | 0.07 | 0.004 | 0.005 | 0.12 |
| **Tobacco smoking** |  |  |  |  |  |  |  |
| Yes | 24(7.7) | 26(8.3) | 33(10.5) | 68(21.7) | 26(8.3) | 0.96±0.08 | 79.11±13.17 |
| No | 114(12.1) | 90(9.5) | 125(13.2) | 302(32.0) | 107(11.3) | 0.94±0.11 | 76.26±14.77 |
| χ 2 /Z | 4.65 | 0.42 | 1.54 | 11.86 | 2.26 | －3.45 | －3.05 |
| P | 0.03 | 0.52 | 0.21 | 0.001 | 0.13 | 0.001 | 0.002 |
| **Alcohol consumption** |  |  |  |  |  |  |  |
| Yes | 24(7.2) | 26(7.8) | 31(9.3) | 74(22.2) | 30(9.0) | 0.96±0.09 | 79.52±13.39 |
| No | 114(12.3) | 90(9.7) | 27(13.7) | 296(32.0) | 103(11.1) | 0.94±0.11 | 76.04±14.69 |
| χ 2 /Z | 6.67 | 1.21 | 4.45 | 11.53 | 1.22 | －3.967 | －4.09 |
| P | 0.01 | 0.29 | 0.035 | 0.001 | 0.27 | ＜0.001 | ＜0.001 |
| **Chronic diseases** |  |  |  |  |  |  |  |
| Yes | 70(11.8) | 57(9.6) | 89(15.0) | 198(33.3) | 73(12.3) | 0.94±0.10 | 75.11±14.49 |
| No | 68(10.2) | 56(8.9) | 69(10.4) | 172(25.9) | 60(9.0) | 0.95±0.10 | 78.63±14.20 |
| χ 2 /Z | 0.77 | 0.19 | 6.02 | 8.34 | 3.51 | －3.50 | －4.57 |
| P | 0.38 | 0.66 | 0.01 | 0.004 | 0.061 | ＜0.001 | ＜0.001 |
| **annual income** |  |  |  |  |  |  |  |
| ≤3000 | 35(14.8) | 23(9.7) | 42(17.8) | 84(35.6) | 36(15.3) | 0.93±0.10 | 76.50±14.90 |
| ＞3000 | 103(10.1) | 93(9.1) | 116(11.4) | 286(28.0) | 97(9.5) | 0.95±0.10 | 77.07±14.33 |
| χ 2 /Z | 4.43 | 0.09 | 7.26 | 5.35 | 6.74 | －3.10 | －0.323 |
| P | 0.04 | 0.76 | 0.007 | 0.02 | 0.009 | 0.002 | 0.75 |
| **Activity time** |  |  |  |  |  |  |  |
| ＜2h | 64(17.2) | 56(15.0) | 73(19.6) | 140(37.5) | 55(14.7) | 0.92±0.12 | 75.17±15.54 |
| ≥2h | 74(8.4) | 60(6.8) | 85(9.6) | 230(26.0) | 78(8.8) | 0.96±0.09 | 77.72±13.88 |
| χ 2 /Z | 20.79 | 21.25 | 23.73 | 16.85 | 9.77 | －5.70 | －2.80 |
| P | ＜0.001 | ＜0.001 | ＜0.001 | ＜0.001 | 0.002 | ＜0.001 | 0.005 |
| **BMI** |  |  |  |  |  |  |  |
| Underweight | 3(7.7) | 5(12.8) | 4(10.3) | 10(25.6) | 5(12.8) | 0.95±0.10 | 76.41±14.95 |
| Normal | 42(10.3) | 30(7.4) | 42(10.3) | 114(28.1) | 48(11.8) | 0.95±0.11 | 76.92±15.72 |
| Overweight | 64(10.2) | 64(10.2) | 81(13.0) | 177(28.3) | 61(9.8) | 0.95±0.10 | 77.87±13.87 |
| Obesity | 29(15.4) | 17(9.0) | 31(16.5) | 69(36.7) | 19(10.1) | 0.94±0.11 | 74.18±12.94 |
| χ 2 /Z | 4.75 | 3.01 | 4.60 | 5.79 | 1.54 | 7.93 | 12.07 |
| P | 0.19 | 0.39 | 0.19 | 0.12 | 0.67 | 0.05 | 0.007 |
| **Nutrition** |  |  |  |  |  |  |  |
| Health | 84(8.3) | 63(6.2) | 104(10.3) | 261(25.8) | 97(9.6) | 0.96±0.82 | 77.22±14.01 |
| Malnutrition | 54(21.8) | 53(21.4) | 54(21.8) | 109(44.0) | 36(14.5) | 0.90±0.15 | 75.93±16.05 |
| χ 2 /Z | 36.92 | 54.48 | 23.88 | 31.45 | 5.08 | －6.60 | －0.86 |
| P | ＜0.001 | ＜0.001 | ＜0.001 | ＜0.001 | 0.02 | ＜0.001 | 0.39 |
| **Cognition** |  |  |  |  |  |  |  |
| Health | 76(8.1) | 59(6.3) | 83(8.8) | 245(26.0) | 87(9.2) | 0.96±0.09 | 78.44±14.75 |
| MCI | 62(19.7) | 57(18.2) | 75(23.9) | 125(39.8) | 46(14.6) | 0.92±0.13 | 72.55±12.46 |
| χ 2 /Z | 32.99 | 39.88 | 48.88 | 21.79 | 7.36 | －7.12 | －7.86 |
| P | ＜0.001 | ＜0.001 | ＜0.001 | ＜0.001 | 0.007 | ＜0.001 | ＜0.001 |
